# AN INTEGRATED TRANSITIONAL CARE INTERVENTION IMPROVES PATIENT-REPORTED OUTCOMES IN OLDER ADULTS WITH STROKE

**DOI:** 10.1093/geroni/igad104.2091

**Published:** 2023-12-21

**Authors:** Maureen Markle-Reid, Kathryn Fisher, Rebecca Ganann, Chris Pollard, Carly Whitmore

**Affiliations:** McMaster University, Hamilton, Ontario, Canada; McMaster University, Hamilton, Ontario, Canada; McMaster University, Hamilton, Ontario, Canada; Hotel Dieu Shaver Health & Rehabilitation Centre, St. Catherines, Ontario, Canada; Centre for Addiction and Mental Health, Hamilton, Ontario, Canada

## Abstract

The transition between hospital and home is associated with significant challenges resulting in hospital readmissions and poor health outcomes. This study aimed to test, in real-world clinical practice, the effectiveness of a Transitional Care Stroke Intervention (TCSI) compared to usual care on patient-reported outcomes and health and social service use costs in older adults (> 55 years) with stroke and multimorbidity (> 2 chronic conditions). The TCSI was delivered virtually by an interprofessional (IP) team, and included care coordination/system navigation support, phone/video visits, monthly IP team conferences, and an online resource co-designed with patient partners to support self-management and system navigation. The primary outcome was risk of hospital readmission after six-months. Secondary outcomes included physical and mental functioning, stroke self-management, patient experience, and health and social service costs. Ninety participants were enrolled (44 intervention, 46 control); 11 (12%) participants were lost to follow-up, leaving 79 (39 intervention, 40 control). Differences favoring the intervention group were seen in physical functioning (SF-12 PCS mean difference: 5.10; 95% CI: 1.58-8.62, p=0.005), self-management (Southampton Stroke Self-Management Questionnaire mean difference: 6.00; 95% CI: 0.51—11.50, p=0.03), and patient experience (Person-Centred Coordinated Care Experiences Questionnaire mean difference: 2.64, 95% CI: 0.81, 4.47, p=0.005). No significant between-group differences were seen for baseline to six-month risk of hospital readmission or total health and social service costs. The TCSI produced greater gains in patient-reported physical health, self-management and experience compared with usual stroke care. These improvements were achieved without increasing total healthcare costs.

